# Physical Stability and Viscoelastic Properties of Co-Amorphous Ezetimibe/Simvastatin System

**DOI:** 10.3390/ph12010040

**Published:** 2019-03-19

**Authors:** Justyna Knapik-Kowalczuk, Krzysztof Chmiel, Karolina Jurkiewicz, Natália T. Correia, Wiesław Sawicki, Marian Paluch

**Affiliations:** 1 Institute of Physics, University of Silesia, SMCEBI, 75 Pułku Piechoty 1a, 41-500 Chorzów, Poland; krzysztof.chmiel@smcebi.edu.pl (K.C.); karolina.jurkiewicz@us.edu.pl (K.J.); marian.paluch@us.edu.pl (M.P.); 2 Univ Lille, CNRS, UMR 8207, UMET, Unité Matériaux et Transformations, F59000 Villeneuve d’Ascq, France; natalia.correia@univ-lille.fr; 3 Department of Physical Chemistry, Medical University of Gdansk, 84-416 Gdansk, Poland; wsawicki@gumed.edu.pl

**Keywords:** ezetimibe, simvastatin, co-amorphous, melt viscosity, solubility enhancement

## Abstract

The purpose of this paper is to examine the physical stability as well as viscoelastic properties of the binary amorphous ezetimibe–simvastatin system. According to our knowledge, this is the first time that such an amorphous composition is prepared and investigated. The tendency toward re-crystallization of the amorphous ezetimibe–simvastatin system, at both standard storage and elevated temperature conditions, have been studied by means of X-ray diffraction (XRD). Our investigations have revealed that simvastatin remarkably improves the physical stability of ezetimibe, despite the fact that it works as a plasticizer. Pure amorphous ezetimibe, when stored at room temperature, begins to re-crystallize after 14 days after amorphization. On the other hand, the ezetimibe-simvastatin binary mixture (at the same storage conditions) is physically stable for at least 1 year. However, the devitrification of the binary amorphous composition was observed at elevated temperature conditions (*T* = 373 K). Therefore, we used a third compound to hinder the re-crystallization. Finally, both the physical stability as well as viscoelastic properties of the ternary systems containing different concentrations of the latter component have been thoroughly investigated.

## 1. Introduction

Cardiovascular diseases (CVDs) are currently the leading cause of death worldwide for both men and women [1,2]. Based on data from the Centers for Disease Control and Prevention (CDC), about 610,000 people in the United States die each year from these diseases. That is roughly the entire population of Washington, D.C., Vermont, or Wyoming. The cornerstone in the prevention of CVDs is lowering high blood cholesterol level [3]. Currently, the first-choice medications for reducing the low-density lipoprotein cholesterol (LDL-C) are statins. They act by inhibiting 3-hydroxy-3-methylglutaryl coenzyme A (HMG-CoA) reductase, thereby blocking cholesterol synthesis.

Among all statins, atorvastatin and simvastatin are considered as the most potent [4,5]. This explains why they can be found on the list of TOP 5 most prescribed drugs [6]. It has to be pointed out that an increase in statins dose offers unfortunately limited LDL-C lowering effect (saturation effect) while at the same time it creates an increased incidence of side effects. Thus, novel active pharmaceutical ingredients (APIs) that might reduce LDL-C levels when co-administered with a statin are of great interest. Ezetimibe is one of such compounds, i.e., a lipid-lowering agent of a new type. In contrast to statins, which suppress the cholesterol synthesis, ezetimibe inhibits intestinal and biliary cholesterol absorption by selectively blocking the Niemann–Pick C1- like 1 (NPC1L1) protein. It has been proven that co-administration of this API with simvastatin is much more effective in reducing mean plasma concentrations of LDL-C than ezetimibe, simvastatin, and also other statins alone [7,8]. As H. E. Bays et al. indicated, the similar reduction in plasma LDL-C level can be achieved with the co-administration of ezetimibe 10 mg plus simvastatin 10 mg as with simvastatin 80 mg alone. In addition, the fixed-dose combination therapy of ezetimibe and simvastatin is well-tolerated by patients [9]. The efficacy and safety of the combined use of both aforementioned APIs have resulted in the appearance of such a combo-product on the market. It is available for oral use as tablets containing 10 mg of ezetimibe and 10, 20, 40, or 80 mg of simvastatin. It is worth mentioning that the commercial forms of ezetimibe and simvastatin exhibit low oral bioavailability, attributed to its poor water solubility. Ezetimibe, which has water solubility is equal to 8.46 ng/L, is characterized by a bioavailability of 35% [10,11]. Simvastatin’s solubility and bioavailability are equal to 1.45 mg/L and 5%, respectively [12]. Since the permeability of both these drugs is high, they are classified to the second class of the Biopharmaceutics Classification System (BCS).

There are several methods that can be employed to increase the solubility and consequently also the bioavailability of APIs from BCS class II [13,14,15]. One of them is based on the preparation of the amorphous form of a chosen compound [16,17,18,19,20]. In the literature, one can find a lot of examples indicating that amorphization remarkably improves the water solubility and bioavailability of poorly soluble drugs [21,22,23,24,25]. It has to be noted that amorphous APIs have unfortunately one disadvantage, which blocks their widespread use [26,27,28,29,30]. Mainly, these APIs are physically unstable. It means that during the time of storage or manufacturing they tend to revert back to their crystalline form [31,32,33,34,35].

In this paper, the binary amorphous composition of ezetimibe and simvastatin in the weight ratio of 1:1 and ternary amorphous compositions containing ezetimibe 1:1 simvastatin and 5, 20, 40 and 60 wt. % of co-polymer Kollidon VA64 were prepared and thoroughly investigated. Thermal properties of both the pure components and the obtained systems were investigated by means of differential scanning calorimetry (DSC). Because the main limitation of widespread use of amorphous pharmaceuticals is physical instability, we thoroughly examined the tendency toward re-crystallization of the investigated system at both standard storage and elevated temperature conditions. The physical stability studies of ezetimibe–simvastatin system were performed by means of X-ray diffraction technique (XRD). Finally, the oscillatory shear rheology was performed to measure the viscoelastic properties of all investigated binary and ternary systems. Based on the obtained rheological data, we plotted the temperature dependences of complex viscosity (η*(T)) of all investigated systems, which can be used for quick determination of the appropriate concentration, temperature, and viscosity for the chosen production method of the ezetimibe–simvastatin system.

## 2. Results and Discussion

### 2.1. Thermal Properties of Ezetimibe, Simvastatin And Its Binary System

**Figure 1:**
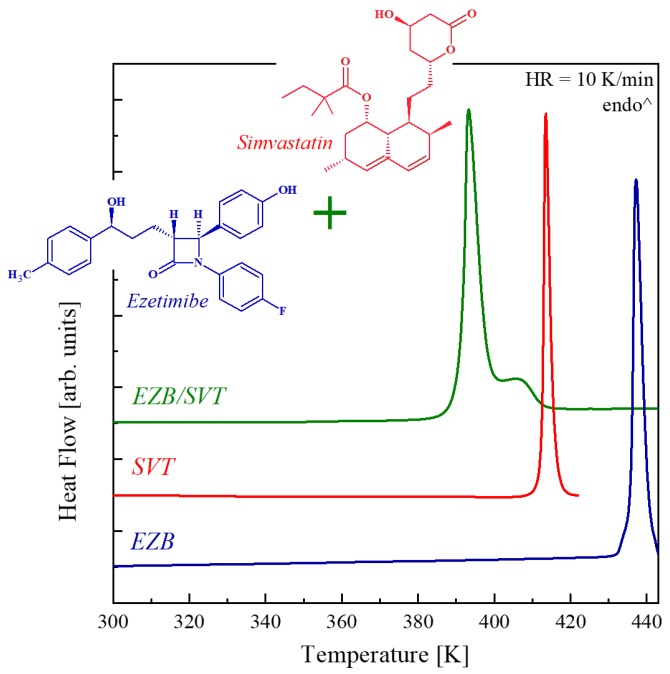
Differential scanning calorimetry (DSC) thermograms of the pure crystalline ezetimibe (EZB; **blue** line), pure crystalline simvastatin (SVT; **red** line), and EZB/SVT physical mixture (**green** line) obtained during heating at 10 K/min.

Figure 1 shows the DSC thermograms obtained during heating of the crystalline ezetimibe (EZB), crystalline simvastatin (SVT), and their physical mixture (EZB/SVT). As can be seen, the DSC traces of pure components reveal only one endothermic peak corresponding to the samples melting. The melting onset of EZB is equal to 436 K, while SVT’s *T_m_* = 412 K. Both these values are in good agreement with the literature data [36,37]. For the physical mixture of EZB/SVT, two thermal effects are observed on the DSC heating curve. The first with the onset at 391 K is related to solidus (eutectic) temperature. The second thermal event with the maximum of the peak at a temperature equal to 406 K and a characteristic tail shape corresponds to the liquidus.

It is worth emphasizing that the observed decrease in the melting points of both EZB and SVT after mixing these two components might be beneficial from the production of the amorphous APIs point of view. Currently, for the manufacturing of amorphous pharmaceuticals, the hot melt extrusion (HME) method is increasingly employed. During this production process, the crystalline API or a drug-polymer composition containing crystalline API is exposed to an elevated temperature in order to melt the system. The lower the extrusion temperature, the safer production process, due to the decreased probability that the sample will undergo thermal decomposition.

**Figure 2:**
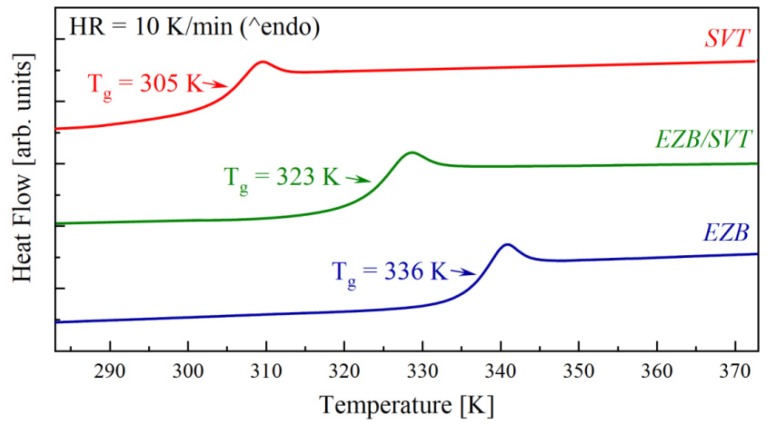
DSC thermograms of quench cooled pure EZB (**blue** line), pure SVT (**red** line), and EZB/SVT composition (**green** line).

Figure 2 presents the second heating run of EZB, SVT, and EZB/SVT, which were performed immediately after melting and quenching the samples in DSC with a rate equal to 10 K/min. As can be seen, the DSC thermograms of freshly melt-quenched samples reveal only one thermal event with a characteristic step-like behavior corresponding to the glass transition. The *T_g_* midpoints of EZB, SVT, and EZB/SVT are equal to 337 K, 305 K, and 323 K, respectively.

Recently, Martinez-Jimenez C. et al. showed that neat amorphous SVT reveals no tendency toward re-crystallization for at least one year when stored at room temperature conditions [38]. Amorphous EZB, however, quickly reverts to its crystalline form when stored at similar conditions. The first sign of re-crystallization of amorphous EZB was detected after only 14 days from vitrification by the melt-quench method [37]. In light of these facts, the physical stability improvement of EZB is needed. By far, the most common approach to suppress the re-crystallization tendency of amorphous APIs is to slow down its molecular mobility (i.e., increase their *T_g_*). This is because the molecular dynamic is believed to be a key factor governing the physical stability of amorphous materials. In EZB/SVT case the opposite situation is observed—mainly the molecular mobility of physically-unstable EZB accelerates after the incorporation of SVT. Consequently, one would speculate that SVT might decrease the physical stability of an amorphous EZB.

### 2.2. Physical Stability Studies of the Amorphous Ezb/Svt System Stored at Both Supercooled Liquid and Glassy State

**Figure 3:**
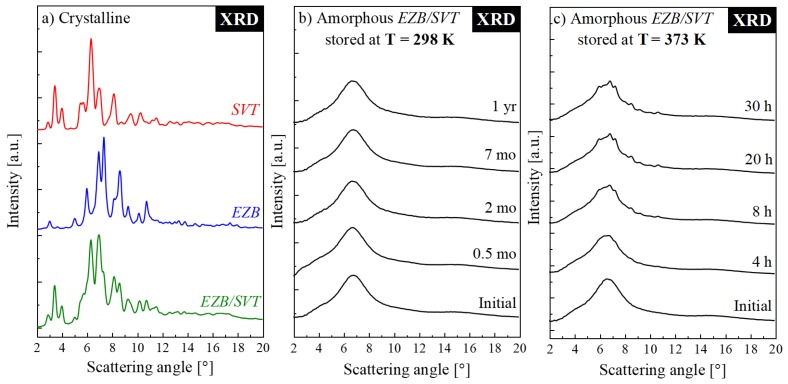
XRD patterns of (**a**) crystalline systems stored at 298 K (**red** line—pure SVT; **blue** line—pure EZB; **green** line—EZB/SVT physical mixture), (**b**) amorphous EZB/SVT system stored at 298 K, (**c**) amorphous EZB/SVT system stored at 373 K.

In order to assess how the SVT changes the physical stability of the amorphous form of EZB, the amorphous EZB/SVT mixture was subjected into two independent XRD experiments. The idea of the first one was to check the mixture’s physical stability at standard storage conditions, i.e., at 298 K and *RH* = 60%. During the second experiment, the sample was stored at elevated temperature conditions (*T* = 373 K). The representative XRD patterns obtained during these experiments are presented, together with the diffractograms of the crystalline EZB, SVT, and EZB/SVT, in Figure 3a–c. It is worth mentioning that the XRD patterns of pure crystalline APIs are in agreement with previous results [38,39]. The samples measured immediately after quenching, as expected, were fully amorphous. The -sharp Bragg’s peaks are not visible on their XRD patterns.

The amorphous form of EZB/SVT composition stored at standard storage conditions was measured by means of XRD at the beginning once daily for a week, later once a week for a month, and then once a month until 1 year. As can be clearly seen even after 1 year, the examined EZB/SVT system did not reveal any sign of re-crystallization. Comparing this result with the previously published by Knapik et al., data for pure EZB, one can conclude that SVT impressively stabilizes EZB. It should be emphasized that herein the improvement of physical stability was achieved despite acceleration the EZB’s molecular mobility. The similar situation was previously observed only in two cases: Nifedipine + nimodipine and simvastatin + nifedipine [39,40]. Such a stabilization effect was explained by the existence of some specific interactions between both drugs. Accordingly, one can expect that, in the EZB/SVT system, the source of the observed stabilization is analogous to the aforementioned cases.

During the second XRD experiment, in which the sample was exposed to the elevated temperature conditions (*T* = 373 K), the XRD patterns were collected every 30 min for 30 h. The representative diffraction patterns are presented in Figure 3c. At this temperature, the first appearance of sharp Bragg’s peaks was noticed after only 210 min. This result indicates that even if the amorphous EZB/SVT system reveals high physical stability when stored below the glass transition temperature, it tends to re-crystallize at a supercooled liquid state. Such a situation is acceptable if for sample manufacturing one would choose a method which does not require sample heating (ex. Spray Drying). It, however, might not be sufficient in cases of production methods involving thermal processing, such as hot melt extrusion or 3D printing.

### 2.3. Impact of KVA 64 Polymer on the Thermal Properties as Well as Physical Stability of the EZB/SVT System

Kollidon VA64 (KVA 64) is a vinylpyrrolidone—a vinyl acetate copolymer. It is amorphous in nature, with a glass transition temperature equal to 378 K and degradation temperature ~500 K [40]. This polymer is well soluble in water and has good processability, and therefore it is commonly used as an excipient for manufacturing of amorphous pharmaceutical products using either solvent (ex. Spray Drying (SD)) or melting (ex. Hot Melt Extrusion (HME)) methods. In such formulations, usually, KVA64 plays a dual role. On the one hand it increases the dissolution rate of the APIs, and on the other hand, it well stabilizes them.

**Figure 4:**
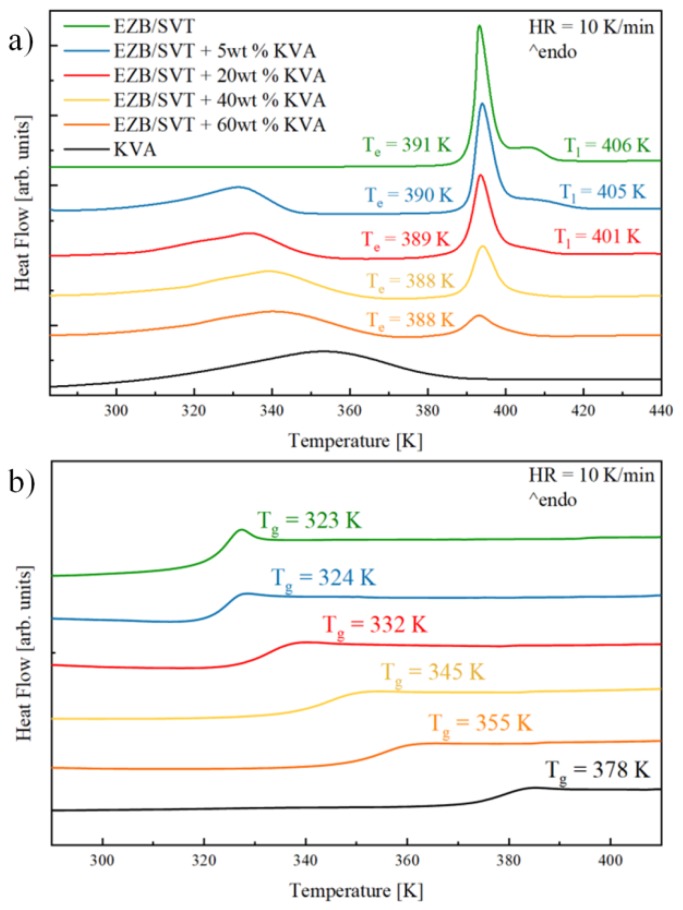
DSC thermograms of (**a**) crystalline and (**b**) amorphous EZB/SVT (**green** lines), EZB/SVT + 5wt. % (Kollidon VA64 (KVA) (**blue** lines), EZB/SVT + 20wt. % KVA (**red** lines), EZB/SVT + 40wt. % KVA (**yellow** lines), EZB/SVT + 60wt. % KVA (**orange** lines) and KVA (**black** lines).

In this section, the impact of KVA64 on the thermal properties and physical stability of a co-amorphous composition containing EZB and SVT is described. Figure 4a presents DSC thermograms of the EZB/SVT physical mixture, neat (as received) KVA 64 polymer, as well as four ternary physical mixtures of EZB, SVT, and KVA in the concentrations: (i) EZB/SVT + 5wt. % KVA, (ii) EZB/SVT + 20wt. % KVA, (iii) EZB/SVT + 40wt. % KVA and (iv) EZB/SVT + 60wt. % KVA. As it has been shown in the first section, the DSC curve of the EZB/SVT crystalline system reveals two thermal events. The first one associated with the eutectic, and the second one reflecting the liquidus. DSC thermogram of the first run of KVA64 reveals only one endothermic event visible in the vicinity of 320–380 K that corresponds to water evaporation. This broad endothermic peak covers the polymer’s glass transition that can be easily noticed at a second DSC heating run (see the black curve in Figure 4b), or (ii) first DSC heating run obtained after sample heating at 373 K for 15 min (data not shown). In the DSC traces of ternary systems (i) and (ii), three thermal endotherms might be noted. First, a very broad thermal event reflecting water evaporation can be found in the vicinity of 320–350 K. A second, with an onset in the neighborhood of 390 K, is connected with the eutectic transition. The third one is visible around 410 K and possesses a characteristic tail shape associated with the liquids phase. On the thermograms of systems (iii) and (iv) the latter endothermic peak becomes invisible, indicating that the ternary eutectic EZB/SVT/KVA was obtained.

Melts of the examined ternary systems have been quenched in DSC with a rate equal to 10 K/min to obtain the amorphous compositions. The glassy samples were subsequently reheated up to *T* = 440 K while obtained thermograms and are presented in Figure 4b. As can be seen, the amorphous mixtures containing EZB, SVT, and KVA are characterized by a single glass transition event which shifts toward a higher temperature with increasing co-polymer content. The presence of a single *T_g_* in binary or ternary compositions usually indicates that these systems are homogenous [41]. If mixtures are not or are only partially miscible, the DSC trace of an amorphous system should reveal multiple *T_g_*s. These can be due to the presence of various drug or polymer rich domains [29]. The values obtained by means of DSC technique, *T_e_*, *T_l_*, and *T_g_*, for all examined samples are presented in Figure 4 and are collected in Table 1.

**Figure 5:**
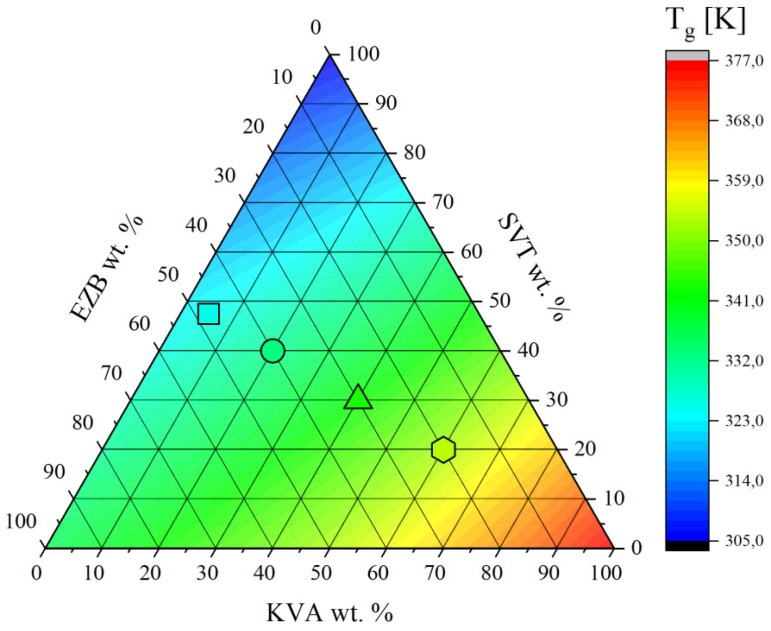
Variation of glass transition temperature in the ternary phase diagram of EZB/SVT/KVA.

Mixing of the multi-components can either be ideal and non-ideal [41,42,43]. The deviation from the ideal mixing in amorphous binary, ternary, or quaternary systems might be examined by comparing the experimental glass transition temperature with the *T_g_* predicted from the Couchman-Karasz (CK) equation given as follows [44,45]:

(1) $$T_{g}=\frac{\sum \Delta C_{pi}x_{i}T_{gi}}{\sum \Delta C_{pi}x_{i}}$$

where *x_i_* is a mass fraction of component *i*, *ΔC_pi_* refers to the change in heat capacity of component *i* between its liquid-like and glassy states, and *T_gi_* denotes a glass transition temperature of component *i*. The similarity in predicted and experimentally determined *T_g_* values strongly suggests ideal mixing, while positive and negative deviation indicates that the mixing is non-ideal [41]. As can be seen in Figure 5 and Table 1, the predicted and experimentally obtained *T_g_* values are in very good agreement indicating that there is good molecular miscibility between all three components, as well as an absence of the intermolecular interactions between them.

**Figure 6:**
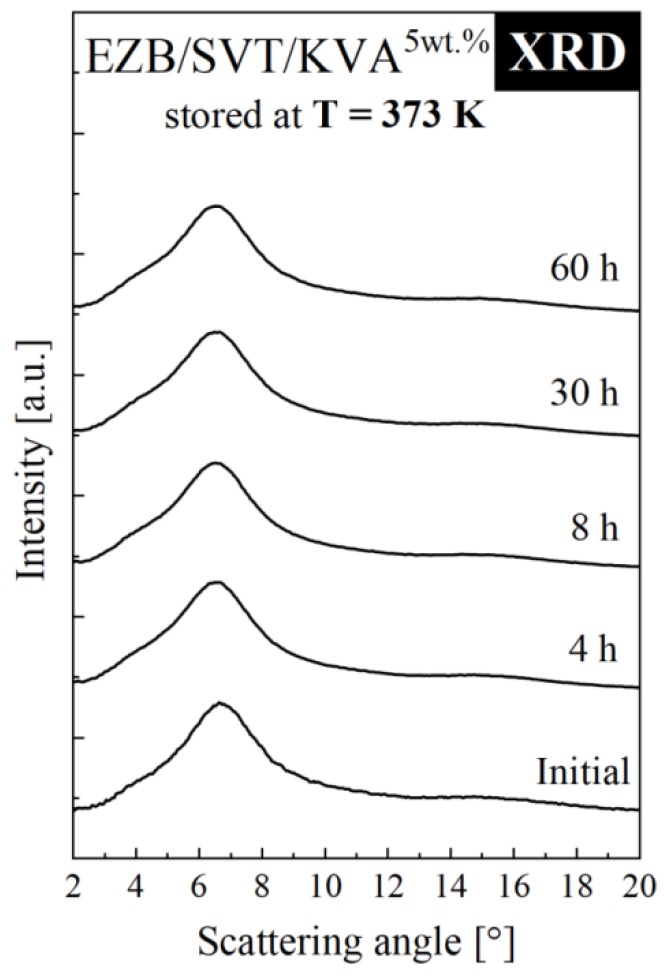
XRD diffraction patterns of ternary amorphous EZB/SVT + 5wt. % KVA measured as a function of storage time at elevated temperature conditions (*T* = 373 K).

To determine how the polymeric additive influx effects the physical stability of EZB/SVT system, the ternary amorphous system containing the lowest amount of the co-polymer has been investigated by means of XRD. The short-term XRD experiment of EZB/SVT + 5wt. % KVA was performed at elevated temperature conditions (*T* = 373 K). During these studies, the mixture’s XRD patterns were collected every 30 min for 60 h. The obtained results are presented in Figure 6. The XRD patterns of both freshly vitrified as well as stored at elevated temperature conditions for 60 h EZB/SVT + 5wt. % KVA mixture reveal a lack of Bragg’s peaks. This indicates that the sample remained amorphous throughout the duration of the experiment. Comparing this result with the data presented in Figure 3c, one can conclude that KVA significantly improves the physical stability of the amorphous EZB/SVT system at elevated temperature conditions (*T* = 373 K).

### 2.4. The Impact of KVA on the Complex Viscosity of the EZB/SVT System

**Figure 7:**
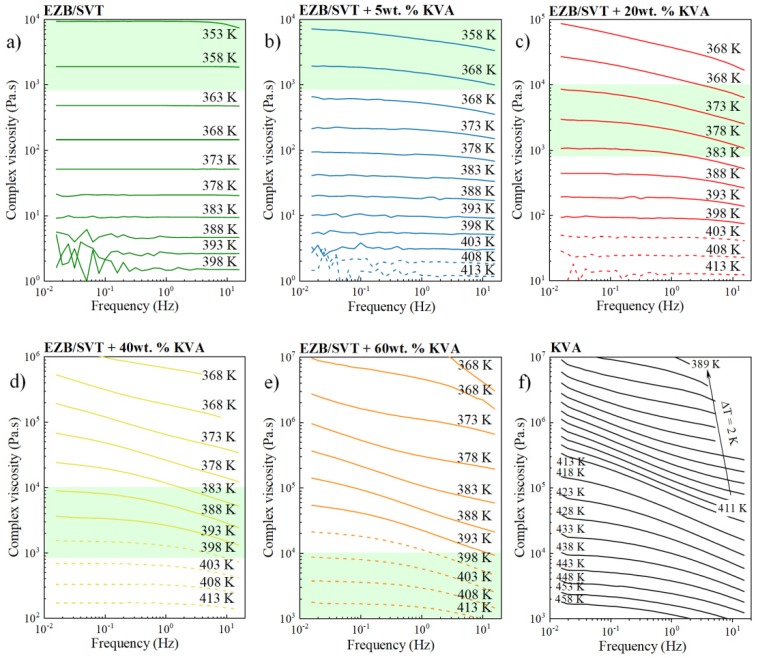
Temperature sweep data showing complex viscosity as a function of frequency of (**a**) the binary amorphous EZB/SVT system, (**b**) the ternary amorphous system of EZB/SVT + 5wt.% KVA, (**c**) the ternary amorphous system of EZB/SVT + 20wt.% KVA, (**d**) the ternary amorphous system of EZB/SVT + 40wt.% KVA, (**e**) the ternary amorphous system of EZB/SVT + 60wt.% KVA and **f**) the neat KVA polymer.

The experiments presented in the previous section proved that even a very small amount of KVA (5wt. %) is able to very effectively suppress the tendency toward re-crystallization of EZB/SVT composition at elevated temperature conditions. It is worth repeating that the physical stability improvement of this binary amorphous drug-drug system is needed only if the chosen production method will involve thermal processing. The best representatives of such methods are hot melt extrusion (HME) or 3D printing (3DP). This kind of manufacturing processes typically includes three stages: (i) Heating and softening of a physical mixture containing API and thermoplastic polymer; (ii) pressurization of molten mass through a die, and (iii) sample solidification, during which the final dosage form is formulated [46,47,48,49,50,51]. To obtain the desired shape of a final product, samples besides high physical stability needs to be characterized by suitable melt viscosity. For example, the small scale extruder required the sample’s melt viscosity at a range of 800–10,000 Pa·s [52]. Thus, in this section, the viscosity of EZB/SVT and its mixtures with KVA have been also investigated. Both the EZB/SVT binary physical mixture as well as the ternary mixtures containing EZB/SVT and KVA polymer have been placed between the fixtures of rheometer and heated up to 413 K. At this particular temperature, the samples were melted and then the gap was set. Next, the material’s complex viscosity was examined in the wide frequency (0.016–15.916 Hz) and temperature (413–353 K) range. The representative complex viscosity curves as a function of frequency are presented in Figure 7.

As can be seen with decreasing the temperature, the samples become more viscous and the viscosity plateau is less pronounced and shifts toward lower frequencies. In this case, the complex viscosity is low, i.e., the sample is more elastic at higher frequencies and increases with decreasing frequency culminating in a consistent viscosity plateau. The lower the polymer content, the less viscous the sample is at constant T, and the more pronounced is the viscosity plateau on the |η*|*(f)*. The shadowed area indicates the generally accepted ‘rule-of-thumb viscosity range’ for small-scale extrusion [52]. The |η*|*(f)* marked by the dashed lines refer to the temperatures higher or equal to the liquidus temperature. As can be seen, EZB/SVT, EZB/SVT + 5wt. % KVA and EZB/SVT + 20wt. % of KVA possess suitable for small scale extruder viscosity value at the temperatures below the liquidus temperature. Therefore, extrusion of such products is impossible. Suitable melting viscosity at T equals or higher than the liquidus temperature can be reached when 40 or higher wt. % of the polymer is employed (see Figure 7d–f).

**Figure 8:**
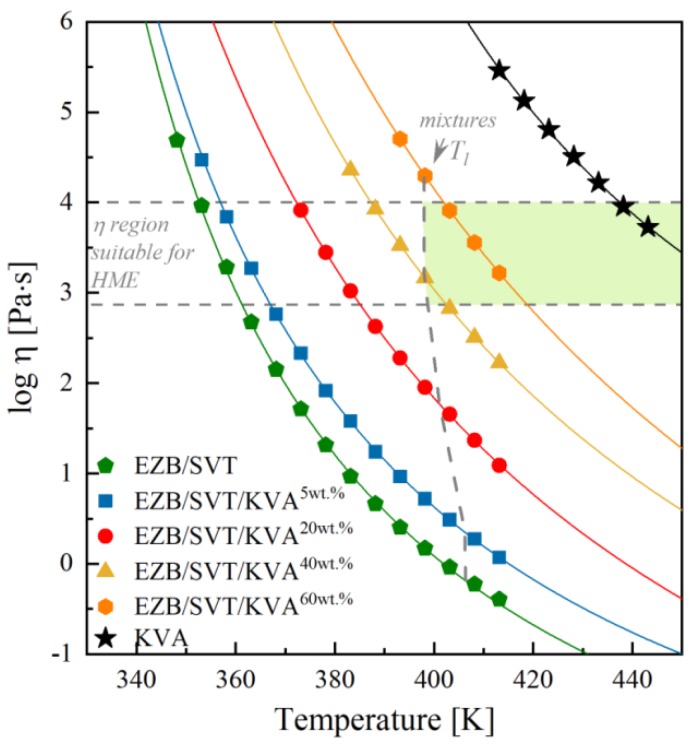
Comparison of the temperature dependences of complex viscosity of KVA (**black** stars), EZB/SVT (**green** pentagons), EZB/SVT + 5wt. % of KVA (**blue** squares), EZB/SVT + 20wt. % of KVA (**red** circles), EZB/SVT + 40wt. % of KVA (**yellow** triangles) and EZB/SVT + 60wt. % of KVA (**orange** hexagons). The solid lines correspond to the VFT fits, dashed horizontal lines denote the upper and the lower range of viscosity suitable for small scale extrusion, and dashed vertical lines represent liquidius temperature of the examined systems, while shadowed area marks the suitable for extrusion region of temperature and viscosity of the EZB/SVT/KVA ternary system.

Figure 8 shows the temperature dependences of viscosity values from the plateau region for all investigated materials, i.e., pure KVA, the binary amorphous system of EZB/SVT, and four ternary amorphous systems containing EZB/SVT and 5, 20, 40, and 60 wt. % of KVA. In order to parameterize the temperature dependence of complex viscosity, we employed the Vogel-Fulcher-Tamman (VFT) equation that is defined as follows [53,54,55]:

(2) $$log_{10}\eta (T)=log_{10}\eta_{\infty }+\frac{DT_{0}}{T-T_{0}}$$

where *T* is temperature and η_∞_, D, *T*_0_ are parameters obtained by fitting Equation (2) to experimentally measured viscosity data. All fitting parameters are collected in Table 2.

Based on Figure 8, where the temperature dependencies of |η*| of all investigated materials are summarized, it is possible to quickly determine both the KVA polymer content and the temperature range, which are suitable to produce physically-stable amorphous EZB/SVT system by means of the melting methods. For example, in the case of small-scale extrusion, where the recommended material’s viscosity range between 800–10,000 Pa·s, the lowest available concentration is equal to 40wt. %. Choosing this particular concentration of the excipient, the minimum temperature needed for sample melting is equal to 398 K. It is worth highlighting that for higher polymer concentrations, higher *T* conditions should be selected (ex. 60wt% of KVA – *T* ≥ 403 K). To better visualize it, we marked in Figure 8 the viscosity area which is appropriate for small scale extrusion and the liquidus temperatures for each examined concentration. Finally, in the shadowed area one can find suitable for extrusion EZB/SVT/KVA compositions and temperatures.

**Figure 9:**
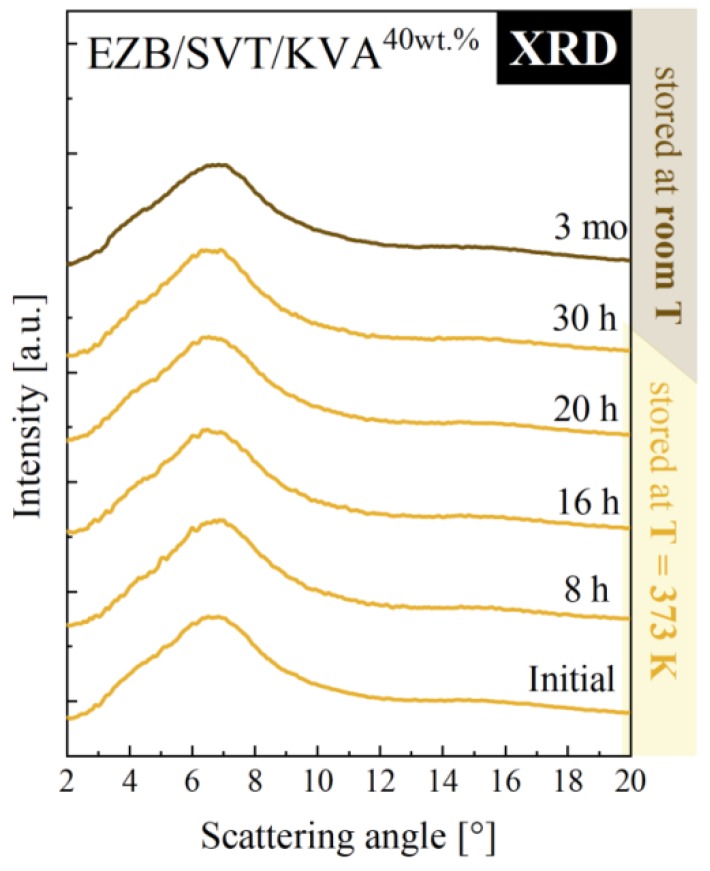
XRD diffraction patterns of ternary amorphous EZB/SVT + 40wt. % KVA measured as a function of storage time. Yellow XRD diffractograms denote the first step of the experiment, during which the sample was stored at elevated temperature conditions (*T* = 373 K), while the brown diffraction pattern corresponds to the second stage of the experiment when the sample was stored at room temperature.

According to the experimental results presented above, in order to produce the EZB/SVT system by means of small-scale extruder, a minimum of 40wt. % of KVA co-polymer should be added. Such a ternary system should be melted at *T* ≥ 389 K. To make sure that *T* = 389 K is indeed high enough to fully amorphize the sample, as well as to investigate whether such a system will be physically stable at both standard storage and elevated temperature conditions, we investigated a quenched from *T* = 398 K EZB/SVT + 40wt. % KVA sample by means of XRD. The representative XRD patterns obtained during this experiment are presented in Figure 9. As can be seen, the diffraction pattern of the freshly prepared sample (quenched from *T* = 398 K) do not reveal sharp Bragg’s peaks, indicating that *T* = 389 K is high enough to fully melt the sample. In the next step of the XRD experiment, the vitrified sample has been heated up to 373 K and stored for 30 h. After each 30 min, the XRD pattern has been registered. As data presented in Figure 9 proves, the investigated EZB/SVT/KVA system was for all that time fully amorphous. The last stage of the XRD measurement consisted in keeping the cooled down to *T* = 298 K ternary composition for a period of 3 months. The sample’s diffraction patterns were collected one a day for a week, next once a week for a month, and finally once a month for 3 months. Data presented in Figure 9 indicates that the examined ternary system is physically stable for at least 3 months after amorphization and annealing at *T* = 373 K for 30 h.

## 3. Materials and Methods

### 3.1. Materials

Ezetimibe drug (EZB) of purity greater than 99% and molecular mass Mw = 409.4 g/mol was purchased from Polpharma (Starogard Gdański, Poland) and used as received. This pharmaceutical is described chemically as ((3R,4S)-1-(4-fluorophenyl)-3-[(3S)-3-(4-fluorophenyl)-3-hydroxypropyl]-4-(4-hydroxyphenyl)azetid in-2-one), with its chemical structure presented in blue inset of Figure 1. Simvastatin drug (SVT) of purity greater than 98% and molecular mass Mw = 418.6 g/mol was purchase from ACOFARMA and used as received. This pharmaceutical is described chemically as Butanoic acid, 2,2-dimethyl-(1S,3R,7S,8S,8aR)-1,2,3,7,8,8a-hexahydro-3,7-dimethyl-8-[2-[(2R,4R) -tetrahydro-4-hydroxy-6-oxo-2H-pyran-2-yl]ethyl]-1-naphthalenyl ester, with its chemical structure presented in the red inset of Figure 1. Kollidon VA64 polymer (KVA) of molecular mass Mw = 45,000–47,000 g/mol was purchased from BASF SE (Germany) and used as received.

### 3.2. Preparation of Binary and Ternary Systems

Both binary amorphous mixtures of EZB and SVT, as well as ternary amorphous systems of EZB/SVT and KVA with different content of the polymer (5, 20, 40, 60wt.% KVA), were prepared by the quench cooling technique. To obtain the homogenous systems prior to the quenching, the physical mixtures were obtained by gentle mixing the appropriate number of samples in a mortal for 15–20 min.

### 3.3. Differential Scanning Calorimetry (DSC)

Thermodynamic properties of EZB, SVT, KVA and their binary and ternary systems were examined using a Mettler–Toledo DSC 1 STARe System. The measuring device was equipped with an HSS8 ceramic sensor having 120 thermocouples. The instrument was calibrated for temperature and enthalpy using indium and zinc standards. Crystallization and melting points were determined as the onset of the peak, whereas the glass transition temperature as the midpoint of the heat capacity increment. The samples were measured in an aluminum crucible (40 µL). All measurements were carried out with a heating rate equal to 10 K/min.

### 3.4. X-ray Diffraction (XRD)

X-ray diffraction experiments were performed for samples stored at both ambient and elevated temperature conditions on a Rigaku-Denki D/MAX RAPID II-R diffractometer (Rigaku Corporation, Tokyo, Japan) with a rotating anode Ag KR tube (λ = 0.5608 Å), an incident beam (002) graphite monochromator, and an image plate in the Debye−Scherrer geometry. The pixel size was 100 µm × 100 µm. Measurements were performed on sample-filled and empty capillaries, to allow subtraction of the background intensity. The beam width at the sample was 0.1 mm. The two-dimensional diffraction patterns were converted into one-dimensional intensity data versus the scattering angle.

### 3.5. Rheological Studies

The viscoelastic properties of neat KVA, the binary system of EZB and SVT, as well as ternary systems containing EZB1:1SVT + different KVA content (5wt%, 20wt.%, 40wt.% and 60wt.%) were measured by means of ARES G2 Rheometer. The instrument was equipped with aluminum parallel plates geometry (diameter = 25 mm). Prior to the oscillation, temperature sweep rheological experiments the samples were placed between the fixtures of rheometer and heated up to 413 K (or *T* = 458 K for neat KVA). At this particular temperature, the examined materials were melted (or liquefied in the case of neat polymer) and then the gap was set at ~1 mm. Next, the material’s complex viscosity was examined in the wide temperature range and frequencies from 0.016–15.916 Hz.

## 4. Conclusions

In this article, the binary amorphous composition of ezetimibe and simvastatin was investigated by means of differential scanning calorimetry, X-ray diffraction, and oscillatory shear rheology. We found that the simple melt-quenching method can be applied to obtain a homogeneous binary system of these two pharmaceuticals. Thermal analysis of the pure crystalline components, as well as physical mixture of ezetimibe and simvastatin, indicated that co-administration of these two APIs leads to melting point depression. The long-term stability studies performed by XRD at standard storage conditions (i.e., *T* = 298 K and *RH* = 60%) have proven that simvastatin, despite the fact that it acts as a plasticizer, remarkably improves the physical stability of amorphous ezetimibe drugs. Unfortunately, the stabilization effect is not enough for the case when the sample is planned to be produced at elevated temperature—at elevated temperature conditions (*T* = 373 K), the re-crystallization of the sample was registered. To overcome this problem, the third component, co-polymer Kollidon VA 64, was proposed as a mixture stabilizer. The physical stability studies performed at elevated temperature conditions indicated that even a small amount of the polymeric additive (5 wt. %) may significantly suppress tendency towards the devitrification of the ezetimibe–simvastatin composition. The first sign of devitrification of the binary amorphous composition noted after 3h and 30 min, while the ternary system containing 5 wt. % of the polymer was physically stable for at least 60 h (when stored at *T* = 373 K).

Finally, the effect of Kollidon VA64 on the viscoelastic properties of the EZB/SVT system in their ternary amorphous compositions was investigated using oscillatory shear rheology. Plots containing temperature dependencies of complex viscosity of all examined systems—constructed based on the rheological data—allowed us to estimate the most suitable, for small scale extrusion, temperature conditions, as well as the polymer concentrations. In order to obtain suitable melt viscosity of the ezetimibe–simvastatin composition, at least 40 wt. % of the polymer is needed. For this particular case, the lowest available temperature is equal to 398 K.

In summary, the binary amorphous ezetimibe–simvastatin system is a very promising candidate for new formulations intended for combined therapy. Apart from the medical and economic benefits of using these APIs together in amorphous forms, such a binary composition provides high physical stability that is essential for further commercial application. For the production of this binary system by melting methods, such as hot melt extrusion or 3D printing, the addition of Kollidon VA64 is required.

## Figures and Tables

**Table 1:** Comparison of *T_e_, T_l_, T_g exp_*, and *T_g pred_* values of pure KVA, the binary mixture of EZB/SVT, as well as the ternary system of EZB/SVT/KVA containing 5, 20, 40 and 60 wt. % of KVA.

System	*T_e_* [K]	*T_l_* [K]	*T_g exp_* [K]	*T_g pred_* [K]
EZB/SVT	391	406	323	323
EZB/SVT + 5wt.% KVA	390	406	324	324
EZB/SVT + 20wt.% KVA	389	401	332	332
EZB/SVT + 40wt.% KVA	388	-	345	342
EZB/SVT + 60wt.% KVA	388	-	355	353
KVA	-	-	378	-

**Table 2:** Comparison of the VFT fitting parameters for neat KVA, the binary mixture of EZB/SVT, and ternary systems of EZB/SVT/KVA containing 5, 20, 40 and 60wt. % of the polymer.

System	*Log*_10_ *η*_∞_	*B = DT*_0_	*T*_0_
EZB/SVT	4.67 ± 0.17	1148.34 ± 60.19	295.12 ± 1.86
EZB/SVT + 5wt.% KVA	−4.55 ± 0.09	1303.53 ± 37.27	290.77 ± 1.22
EZB/SVT + 20wt.% KVA	−6.19 ± 2.67	2411.64 ± 148.98	269.49 ± 3.66
EZB/SVT + 40wt.% KVA	−5.59 ± 0.22	2515.29 ± 126.57	273.42 ± 3.04
EZB/SVT + 60wt.% KVA	−7.15 ± 0.4	3816.61 ± 278.45	253.35 ± 5.37
KVA	−4.13 ± 0.83	2981.70 ± 565.27	278.26 ± 13.87
